# Coping and quality of life of parents of children with achondroplasia—a narrative review

**DOI:** 10.3389/fmed.2025.1500389

**Published:** 2025-05-30

**Authors:** Adekunle Adedeji, Stefanie Witt, Florian Innig, Inês Alves, Chiara Provasi, Marco Sessa, Klaus Mohnike, Julia Quitmann

**Affiliations:** ^1^Department of Social Work, Hamburg University of Applied Sciences, Hamburg, Germany; ^2^Department of Medical Psychology, University Medical Center, Hamburg-Eppendorf, Hamburg, Germany; ^3^Federal Association for People of Short Stature and their Families (Bundesverband Kleinwüchsige Menschen und ihre Familien e.V.), BKMF, Bremen, Germany; ^4^National Patient Organization for Skeletal Dysplasias – ANDO, School of Health and Human Development, University of Évora - CHRC, Évora, Portugal; ^5^Italian Association on Achondroplasia (Associazione per l’Informazione e lo studio dell’acondroplasia) AISAC, Milan, Italy; ^6^Children’s Hospital, Otto-von-Guericke-University, Magdeburg, Germany

**Keywords:** PROMs, patient report, proxy report, quality of life, parents of pediatric patients, achondroplasia, rare disease, HrQOL

## Abstract

**Background:**

Caring for individuals with a chronic disease imposes a substantial burden on parents, significantly impacting their quality of life. For parents of children with achondroplasia, caregiving has notable implications for coping mechanisms and overall wellbeing. This review summarizes findings on these parents’ coping strategies and quality of life.

**Methods:**

A narrative approach was employed to synthesize research on parental outcomes related to caring for a child with achondroplasia. The PRISMA chart flow was utilized to present the article screening strategy and results, following established guidelines for systematic reviews.

**Results:**

The review reveals a scarcity of studies examining the impact of caring for a child with achondroplasia on parental outcomes, with only two studies meeting the inclusion criteria. These studies suggest that having a child with achondroplasia significantly affects parental coping and quality of life, indicating substantial emotional and social implications. Additionally, no specific tools or measures to assess outcomes for these parents, highlighting a significant gap in research and resources.

**Conclusion:**

The parental experience of caring for a child with achondroplasia involves significant emotional and social challenges. Stressors from emotional distress, social isolation, altered family dynamics, and demanding healthcare interactions underscore the need for robust support systems. Addressing the research gaps requires developing and validating specific measures to assess the outcomes for parents of children with achondroplasia accurately. This will encourage further research and guide the development and evaluation of interventions to improve the coping and QoL of parents of children with achondroplasia.

## Background

Caring for individuals with a chronic disease imposes a substantial burden on parents, significantly impacting their quality of life (QOL) (1, 2). Achondroplasia, a rare chronic genetic condition affecting bone growth, is characterized by disproportionate short stature (3). It manifests in approximately 1 in 15,000 to 40,000 live births and typically follows an autosomal dominant inheritance pattern (4, 5).

Achondroplasia is a rare genetic condition caused by mutations in the *fibroblast growth factor receptor 3* (FGFR3) gene, which regulates bone growth and results in abnormal cartilage formation, particularly in the long bones (6). Clinically, it is characterized by disproportionate short stature (with a final adult height approximately −6.0 SDS compared to the general population), shortened limbs, and distinctive craniofacial features such as a prominent forehead and midface hypoplasia (7). Spinal abnormalities are common and include exaggerated inward curvature (lordosis) and, occasionally, outward curvature (kyphosis). Additional musculoskeletal manifestations may include bowing of the legs (genu varum), typically observed in early childhood and often improving over time. Other frequent complications include recurrent ear infections due to Eustachian tube dysfunction and, in rare cases, hydrocephalus resulting from cerebrospinal fluid accumulation (3, 6, 8). Delays in gross motor and ambulatory development are also well-documented in affected children (9). Individuals with achondroplasia face a 10-year shorter life expectancy than the general population, and experience significant disparities in quality of life, as indicated by lower physical and mental health scores compared to the national American population (43, 44).

The medical complexities associated with achondroplasia, including frequent surgeries, routine medical appointments, and the need for ongoing monitoring, demand substantial time and emotional investment from parents and family caregivers (10). This vigilance can induce chronic stress, fatigue, and burnout, adversely affecting overall wellbeing.

Moreover, the necessity to balance caregiving duties with other familial and professional responsibilities often results in diminished leisure time and social isolation (11, 12), exacerbating mental health concerns such as anxiety and depression. Financial strain stemming from medical expenses and potential income loss due to reduced work hours or cessation adds further stress, contributing to diminished QOL for parents (12). Furthermore, beyond the physical and medical aspects, the distinctive features of achondroplasia also activate numerous social challenges in individuals with achondroplasia, which also affect their families (13).

QOL is a multidimensional concept encompassing overall wellbeing, including physical health, psychological state, level of independence, social relationships, personal beliefs, and environmental factors (14, 15). It is evaluated through objective measures such as health status and functional abilities and subjective assessments, including perceptions of happiness, life satisfaction, and fulfillment. The World Health Organization (WHO) defines QOL as an individual’s perception of their position within their cultural and value systems relative to their goals, expectations, standards, and concerns (16). This holistic approach acknowledges that QOL is influenced by various factors beyond the absence of disease or infirmity, emphasizing the importance of a balanced interplay between physical, psychological, and social wellbeing (17). In the context of individuals with achondroplasia, QOL may be profoundly affected by the unique and continuous demands associated with this condition (5). Parents may often endure significant physical and emotional stress as they navigate the complexities of managing health issues as well as the social confrontments/challenges of fear of their kids being bullied (1). This multifaceted strain on physical, emotional, social, and financial aspects significantly impacts the QOL of parents of children and adolescents with achondroplasia.

Moreover, comprehensively understanding the coping strategies employed by parents of children with Achondroplasia may be crucial for assessing the condition’s impact on family dynamics and parental wellbeing. Coping strategies encompass various cognitive, emotional, and behavioral responses individuals employ to manage stressors and adapt to challenging circumstances (18, 19). In the context of parental caregiving for achondroplasia patients, parents may utilize various coping mechanisms to navigate the intricate and demanding responsibilities associated with their child’s medical needs, social interactions, and educational challenges. These strategies may impact the caregiving ability and the QOL of the parent providing care.

### The current review

Examining parent coping and QOL as outcomes provides valuable insights into the efficacy of support systems, interventions, and resources to enhance parental resilience, reduce stress, and promote family cohesion in providing care for chronic disease patients (20, 21). The current review is rooted in the unique and substantial challenges parents of children with Achondroplasia face as primary caregivers and the significant medical, physical, and social complexities surrounding Achondroplasia. Despite the critical role these parents play in managing their child’s condition and the profound effects on their wellbeing, there is a lack of consolidated research examining their coping mechanisms and QOL. Reviewing this outcome in this population is essential to gain a deeper understanding of how parents manage challenges and the effectiveness of their coping strategies. Such a review would provide valuable insights into the existing tools used to assess parental coping and QOL, identify gaps in current research, and highlight areas needing further investigation. Moreover, it would inform healthcare providers, policymakers, and support organizations about the specific needs of these families, leading to the development of targeted interventions and support systems.

## Materials and methods

This review aims to summarize available findings on the coping and quality of life of parents of children with Achondroplasia. The methodological framework follows established guidelines for conducting and analyzing systematic reviews (22). The PRISMA 2020 flowchart presented search and screening results (23).

### Inclusion and exclusion criteria

The inclusion criteria (IC) and exclusion criteria (EC) were established using the PICOS format (22), as detailed in Table 1. The study focused on research involving parents of children with Achondroplasia. Consequently, studies involving the patients themselves were excluded (EC 1).

**Table 1:** Inclusion and exclusion criteria based on the PICOS scheme.

Components	Description
Patient	
IC 1	Parents of individuals with achondroplasia
EC 1	Individuals with Achondroplasia
Intervention	
IC 2	Quantitative evaluation of parent outcomes
EC 2	Non validated measures
*Comparator*	
	Not relevant
*Outcome*	
IC 3	Coping and Quality of Life
EC 3	–
Publication	
IC 4	Original Studies published in peer-reviewed journals with abstract, title and full text in English or German
EC 4.1	Unpublished Studies, book chapters, congress contributions, Clinical reports

IC = Inclusion Criteria; EC = Exclusion Criteria.

Additionally, the selected studies had to examine parent outcomes using validated Patient-Reported Outcome Measures (PROMs) or Observer-Reported Outcome Measures (ObsROMs) (IC 2). Inclusion also required that the studies address at least one outcome related to the parent’s quality of life or coping (IC 3). Only original studies published as peer-reviewed journal articles in German or English, with abstracts, titles, and full texts available, were considered (IC 4), with no date restrictions applied. Unpublished studies, book chapters, conference contributions, and case reports were excluded (EC 4.1).

### Information sources and search strategy

Comprehensive searches were conducted in PubMed and the Web of Science to identify relevant literature, encompassing publications up to 17 January 2024. Preliminary searches were conducted before initiating the formal search process. The final search string, as detailed in Table 2, was employed in the PubMed databases and the Web of Science. The search string covers (BLOCK A) AND (BLOCK B) AND ((BLOCK C PART I) OR (BLOCK C PART II)) (see Table 2).

**Table 2:** Terms used in systematic database literature search.

Category	Search string in English
Block A: Achondroplasia-related terms (90.696 Results; 17.01.2024)	(((((((((achondroplasia[MeSH Terms]) OR (achondroplasia[Title/Abstract])) OR (Rare Diseases[MeSH Terms])) OR (rare disease*[Title/Abstract])) OR (Skeletal Dysplasia[Title/Abstract])) OR (Bone Diseases, Endocrine[MeSH Terms])) OR (dwarfism[MeSH Terms])) OR (dwarfism[Title/Abstract])) OR (Short stature[Title/Abstract])) OR (orphan disease*[Title/Abstract])
BLOCK B: parent focus (2.175.644 Results; 17.01.2024)	((((((((((((Parents[MeSH Terms]) OR (parent*[Title/Abstract])) OR (caregiv*[Title/Abstract])) OR (caregivers[MeSH Terms])) OR (mothers[MeSH Terms])) OR (fathers[MeSH Terms])) OR (mother*[Title/Abstract])) OR (father*[Title/Abstract])) OR (family[MeSH Terms])) OR (famil*[Title/Abstract])) OR (carer*[Title/Abstract])) OR (foster*[Title/Abstract])) OR (guardian*[Title/Abstract])
BLOCK C: PART I – QOL/STRESS/BURDEN (1.125.609 Results; 17.01.2024)	(((((((((((((((((((Quality of life[MeSH Terms]) OR (Health-related quality of life[MeSH Terms])) OR (patient reported outcome measures[MeSH Terms])) OR (quality of life assessment[Title/Abstract])) OR (Quality of Life[Title/Abstract])) OR (Health-related Quality of life[Title/Abstract])) OR (patient reported outcome measures[Title/Abstract])) OR (Quality of life survey[Title/Abstract])) OR (quality of life questionnare[Title/Abstract])) OR (quality of life tool[Title/Abstract])) OR (Mental health[Title/Abstract])) OR (Mental health[MeSH Terms])) OR (Psychological Well-being[MeSH Terms])) OR (psychological well-being[Title/Abstract])) OR (caregiver burden[MeSH Terms])) OR (caregiver burden[Title/Abstract])) OR (Stress, Psychological[MeSH Terms])) OR (parental Stress[Title/Abstract])) OR (burden*[Title/Abstract])) OR (family stress[Title/Abstract])
BLOCK C: PART II – COPING (628.036 Results; 17.01.2024)	(((Coping[Title/Abstract]) OR (Deal*[Title/Abstract])) OR (Adaptation, Psychological[MeSH Terms])) OR (psychology, applied[MeSH Terms])

### Study selection

**Figure 1:**
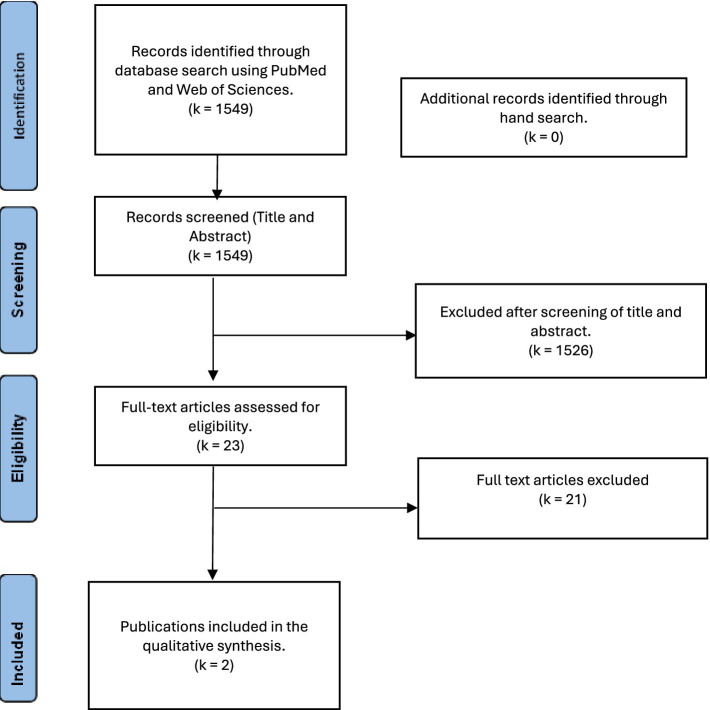
PRISMA-flow-diagram for the presentation of the study selection process (23).

The PubMed and Web of Science databases were searched, identifying *k* = 1,549 publications. Two independent researchers screened the titles and abstracts of the studies. Records not meeting the inclusion criteria were excluded (*k* = 1,526). Two independent researchers screened the remaining *k* = 23 full texts to assess eligibility. At the end of the study selection process, *k* = 2 records were included in the review. Figure 1 shows the study selection process for this systematic review in the PRISMA flow chat.

### Screening and study selection

The identified studies were exported to Excel after the systematic search. Subsequently, two independent reviewers thoroughly screened all titles and abstracts, adhering to the predefined inclusion and exclusion criteria. Excluded references were annotated with the specific reason for exclusion. Articles marked as “excluded” by both reviewers were eliminated from consideration. In cases where conflicting votes (e.g., ineligible vs. potentially or probably eligible) were present, the reviewers engaged in discussions until a consensus was reached.

### Data extraction and synthesis

Data were extracted from the included publications using an Excel sheet. The extracted data included study characteristics (e.g., study design, sample size), parent characteristics (e.g., age, marital status), and measures of QoL or Coping. The extracted data were synthesized and presented in a narrative format. The results were summarized according to the results and patterns presented in the identified publications.

### Quality assessment

The quality of the included studies was assessed using the Joanna Briggs Institute (JBI) Critical Appraisal Checklist (24). The tool assesses the methodological quality of studies reporting prevalence data, such as cross-sectional studies and surveys. The checklist includes nine criteria assessing the appropriateness of the sample frame and sampling strategy and a description of participants and settings. By systematically examining these components, the JBI checklist facilitates a rigorous evaluation of the validity and quality of prevalence studies, aiding reviewers in systematic reviews and evidence synthesis. As shown in Table 3, the results of this evaluation suggest that one of the studies has high quality while the other is of low quality.

**Table 3:** Adapted rating of the methodological quality of included studies (*k* = 2) based on Joanna Briggs Institute (JBI) Critical Appraisal Checklist.

	Author	Clear RQ^~^	Appropriate sample frame^1^	Sampling method^2^	Sample size^3^	The subject and setting adequately described^4^	Data analysis has sufficient coverage^5^	Condition identification^6^	Condition measured^7^	Appropriate statistical analysis^8^	Response rate adequate^9^	Overall quality
1	Silibello et al. (25)	+	+	+	+	+	+	+	-	+	-	High
2	Witt et al. (26)	+	+	+	+	+	+	+	-	+	-	High

Are there clear research questions (RQ)? 1 Was the sample frame appropriate for the target population? 2 Were study participants sampled appropriately? 3 Was the sample size adequate? 4 Were the study subjects and the setting described in detail? 5 Was the data analysis conducted with sufficient coverage of the identified sample? 6 Were valid methods used to identify the condition? 7 Was the condition measured in a standard, reliable way for all participants? 8 Was there appropriate statistical analysis? 9 Was the response rate adequate, and if not, was the low response rate managed appropriately? + quality criterion met, − quality criterion not met, 0 = not enough information available overall assessment of methodological study quality: HIGH = both screening questions and all quality criteria met, MEDIUM = both screening questions met, and 1–2 quality criteria not met/not enough information available, LOW = at least one screening question not met or more than 2 quality criteria not met/not enough information available.

## Results

### Study characteristics

The current review analyses two studies published in 2016 and 2019, sourced from Italy and Germany. The first article by Silibello et al. (25) focused on daily changes and coping among 154 families with a child suffering from a rare disability registered at a public center for rare diseases in Northern Italy. They describe the socio-demographic characteristics, health problems, and living conditions of families with children suffering from rare genetic diseases, including a subsample of seven parents of children with achondroplasia. The data were collected between September 2011 and April 2013. No descriptive data on the parents of children with Achondroplasia were reported in the article.

The second article, published by Witt et al. (26), focused on evaluating the quality of life of children with achondroplasia and the quality of life of their parents. The study used cross-sectional data and was part of a more extensive study, the Achondroplasia Personal Life Scale Experience Scale study. Families (one parent per child) of children with Achondroplasia were recruited through the German Association for People of Short Stature and their Families. Only families with a clinical diagnosis of achondroplasia and no other known serious illnesses were included in the study. A total of 73 families participated in the study. The children’s ages ranged from 5 to 14. About 77% of the parent participants were female. No further demographic data was reported for the parent.

### Patient reported outcome measures

#### Coping: brief-COPE

Silibello et al. (25) use the Brief-COPE questionnaire to assess coping as a parent outcome. The 28-item questionnaire assesses the personal reactions of the parents concerning the commitment required by the child’s illness. The Brief-COPE, first ideated by Carver (27) is a reduced version of the COPE Inventory by Carver et al. (28). The scale assesses three domains: Problem-Focused Coping, Emotion-Focused Coping, and Avoidant Coping. Furthermore, the study reported the following facets of coping: self-distraction, Denial, Substance Use, Behavioral disengagement, Emotional Support, Venting, Humor, Acceptance, Self-Blame, Religion, Active Coping, Use of Instrumental Support, Positive Reframing, and Planning. The generic scale is validated and reliable for the different subdomains among different populations and has been used among caregivers of different chronic or rare diseases (see Table 4).

**Table 4:** Description and characteristics of reviewed studies (*n* = 2).

Author	Year	Title	Diagnosis	PROM	Scales/domains	Number of items	Cronbach’s alpha (reliability)	Report-form (PRO/ObsRO)	Age-groups of PROM	Original language
G. Silibello; P. Vizziello; M. Gallucci; A. Selicorni; D. Milani; P. F. Ajmone; C. Rigamonti; S. De Stefano; M. F. Bedeschi; F. Lalatta	2016	Daily life changes and adaptations were investigated in 154 families with a child suffering from a rare disability at a public center for rare diseases in Northern Italy	Rare diseases, including Achondroplasia	Brief-COPE	- Problem-Focused Coping - Emotion-Focused Coping - Avoidant Coping	28	Excellent for emotion-focused, problem-focused, and dysfunctional coping strategies among caregivers of individuals with dementia (alpha from 0.72–0.84)	PRO	Adults General population Patient Group	English
S. Witt; B. Kolb; J. Bloemeke; K. Mohnike; M. Bullinger; J. Quitmann	2019	Quality of Life of children with Achondroplasia and their parents’ German cross-sectional study	Achondroplasia	SF-8	2 Summary measures: - physical health summary - Mental health summary	8	Physical component = 0.86 mental component = 0.85	PRO	Adults General population Patient Group	English

#### Quality of life: SF-8

Witt et al. (26) measured parents’ quality of life using the Short-Form-8 questionnaire (SF-8). The questionnaire is a short form of the SF-36, a generic measure of quality-of-life instrument adopted in different studies and across different demographic and patient populations.

The questionnaire captures overall health, physical functioning, physical role, bodily pain, vitality, social functioning, mental health or emotional problems, and emotional role. The eight items are categorized into two summary measures: physical health summary and mental health summary. In the current sample, the total scores of the SF-8 showed good reliability for the physical component score with Cronbach’s *α* = 0.86 and the mental component score with Cronbach’s *α* = 0.85.

The included studies highlighted significant implications of the child’s health condition on parental coping strategies and subjective quality of life, underscoring the broader impact of achondroplasia on families. Both studies used validated instruments to assess parental outcomes despite being generic and applicable to various sociodemographic and patient populations. Witt et al. (26) focused on the QoL of parents using a standard QoL measure, while Silibello et al. (25) investigated specific coping strategies through detailed demographic analysis. Together, these studies highlight the multifaceted challenges faced by parents of children with achondroplasia and underscore the critical need for comprehensive support systems that address both mental and physical health aspects for the entire family.

### The findings

Silibello et al. (25) conducted a comprehensive study to explore how various factors influence the coping strategies of parents with children requiring significant care. Their findings revealed interesting associations between parental coping mechanisms and specific demographic variables. Notably, they found that the child’s gender was linked to the use of “Positive Reframing” by mothers, suggesting that mothers may adopt more optimistic reinterpretations of situations based on the gender of their child. Additionally, the child’s age was correlated with the coping strategy of “Acceptance,” indicating that as children grow older, parents may be more inclined to accept the circumstances surrounding their child’s condition.

Silibello et al. (25) also highlighted parental education levels’ impact on coping strategies. For both mothers and fathers, higher education levels were negatively associated with “Behavioral Disengagement,” implying that more educated parents are less likely to disengage behaviorally in response to stress. Conversely, mothers with higher education tended to use “Self-Distraction” more frequently, while fathers with higher education levels were less likely to employ “Positive Reframing.” These findings suggest that education influences how parents manage stress and reframe their experiences. Furthermore, the research indicated that the number of children in the family played a significant role in coping strategies. Families with more children were more likely to resort to “Self-Distraction,” “Denial,” and “Self-Blame,” highlighting the increased stress and complexity of managing multiple children, especially when one requires special care. Additionally, the strategy of “Planning” was positively correlated with the couple’s marital status, suggesting that married couples might be more inclined to engage in forward-thinking and structured problem-solving.

Considering these findings, Silibello et al. (25) concluded that the Italian healthcare system should prioritize investing in operator training and promoting family-centered care (FCC). They emphasized the importance of equipping healthcare providers with the skills to support these families effectively. The researchers also underscored the complexity of managing the network of health and social services necessary for families dealing with chronic health conditions in their children, advocating for a more integrated and supportive approach to care.

Similarly, Witt et al. (26), in a comparative analysis assessing the QoL scores of parents of children with achondroplasia against normative values from a German reference population, found significantly lower scores for the mental component of QoL among the parents. In contrast, physical QoL scores showed no significant differences. Further analysis revealed that parental physical and mental QoL were significant predictors of the parent-reported QoL of their children.

Witt et al. (26) concluded that the results underscore the necessity of addressing patients’ and parents’ QoL in clinical settings. The study suggests that chronic health conditions such as achondroplasia can impact the daily lives of entire families as they adapt to the child’s unique needs. The authors argue that reduced mental QoL in parents exacerbates the overall burden imposed by the child’s chronic condition. Consequently, since parental QoL significantly influences the perceived QoL of the children, clinicians must observe and care for parents’ QoL beyond the children. This highlights the importance of considering the psychosocial circumstances of the entire family in managing chronic health conditions.

## Discussion

### Summary of key findings

1. *Significant Implications*: Existing research suggests that having a child with achondroplasia significantly affects parental outcomes, indicating substantial emotional and social implications for the parents.
2. *Limited Research on Parental Outcomes:* There is a scarcity of studies examining the impact of caring for a child with achondroplasia on parental outcomes.
3. *Lack of Specific Measures:* No specific tools or measures are currently designed to assess the outcomes for parents of children with achondroplasia, highlighting a gap in the available research and resources.

The current review summarizes findings on parental outcomes associated with caring for children with achondroplasia. The findings suggest that having a child with achondroplasia significantly affects parental outcomes, indicating substantial emotional and social implications for the parents. This impact can be multifaceted, encompassing psychological stress, changes in family dynamics, and broader social interactions. These findings are supported by other studies that assess parent outcomes linked with the impact of providing care for children with chronic diseases (29, 30). Cohn et al. (29), in a systematic review, found that parents of children with chronic illness often experience heightened emotional stress due to the chronic nature of the condition and the associated health complications. Further studies suggest that these parents frequently report feelings of anxiety, depression, and helplessness (31, 32). The persistent need for medical interventions, coupled with concerns about their child’s future, contributes to this emotional burden. While many of the emotional, social, and psychological challenges faced by parents, such as initial grief, uncertainty about the future, and navigating medical systems, are common across various chronic and rare conditions, achondroplasia presents distinctive features. These include the visibility of the condition due to short stature, potential stigmatization, and a unique clinical trajectory involving specific surgical and orthopedic interventions. Such characteristics may intensify the parental burden and shape coping differently compared to less visible or differently progressing conditions. Parents of achondroplasia patients may worry about their child’s physical development, potential for bullying, and the ability to lead an independent life (33). These worries can lead to stress, affecting their overall mental health and wellbeing.

Similarly, research suggests that parents of children with chronic diseases often encounter isolation and stigma (34, 35). This may be more so in cases where the condition is visible and can lead to intrusive questions and unwelcome attention from strangers. This attention can be distressing. Additionally, parents might find it challenging to relate to other parents who do not share similar experiences, leading to social withdrawal (34). For parents of achondroplasia patients, support networks, both formal and informal, become crucial in mitigating these effects. Research indicates that strong social support can buffer against the negative emotional impacts, providing parents with a sense of community and shared understanding (36). Furthermore, interacting with healthcare systems presents another layer of complexity. Parents often need to advocate for their child’s needs, which can be exhausting and time-consuming. The need for specialized care and the potential for frequent medical appointments can disrupt daily life and work schedules, adding to the stress experienced by parents.

Similar to the findings from Silibello et al. (25), other studies on accessing outcomes of chronically ill children suggest that many parents develop effective coping mechanisms and display remarkable resilience over time (37, 38). This is attributed to online and offline engagement in support groups and the emotional support and practical advice from such engagement (39). Similarly, educational programs that enhance understanding of achondroplasia and its management are expected to empower parents, helping them feel more in control and less overwhelmed (10).

The process and findings of the current review also highlight the scarcity of research examining the impact of caring for a child with achondroplasia on parental outcomes. This paucity of literature underscores a significant gap in understanding these families’ full spectrum of challenges and needs. The limited number of studies focused on this area indicates that the unique experiences of parents caring for a child with achondroplasia may be underrepresented in the broader discourse on disability and caregiving. This can contribute to the lack of awareness and implicate policy and practice Gaps (40). Without sufficient research, there is a risk of underestimating the emotional, social, and psychological toll on parents. This lack of awareness can lead to inadequate support systems and resources being made available to these families. Similarly, policymakers and healthcare providers rely on robust research to inform their decisions and practices. The dearth of studies means that there may be gaps in policies and practices that specifically address the needs of families dealing with achondroplasia, leading to insufficient or misdirected support.

This scarcity of research is further linked to the unavailability of specific measures for parental outcomes when having a child with achondroplasia. The review identifies a critical gap in the available research and resources: the absence of specific tools or measures designed to assess the outcomes for parents of children with achondroplasia. This lack of tailored assessment instruments implies inadequate assessment, limited comparability, and a hindrance to intervention development and evaluation (41, 42). Generic measures of parental quality of life and coping may not fully capture the unique challenges faced by parents of children with achondroplasia. This can lead to an incomplete or inaccurate understanding of their experiences and needs. Furthermore, comparing findings across different studies or populations is challenging without specific tools. This limits the ability to draw general conclusions or identify patterns and trends specific to this group. The lack of precise assessment tools hinders the development of effective interventions. Designing and evaluating targeted support programs becomes difficult without clearly understanding these parents’ specific outcomes and needs.

## Conclusion and future research

Raising a child with achondroplasia presents parents with considerable emotional and social challenges. These include persistent psychological stress, social isolation, shifts in family dynamics, and complex interactions with healthcare systems. To better understand and support these experiences, there is a pressing need for the development and validation of condition-specific assessment tools. Such instruments should comprehensively capture key domains of parental outcomes, including mental health, coping strategies, family relationships, and the financial and occupational burden associated with caregiving.

Integrating these insights into clinical practice requires routine psychosocial screening at critical developmental milestones, such as diagnosis, early childhood, and school entry. Clinicians should be supported with tailored screening instruments and clear referral pathways to psychological services and peer support networks. Additionally, providing anticipatory guidance on foreseeable medical and social challenges can help families navigate their journey with greater confidence and resilience.

Future research should adopt large-scale, quantitative approaches to generate a more robust understanding of the parental experience and to validate the proposed assessment tools. Furthermore, the impact of achondroplasia on siblings remains an underexplored area. Studies examining sibling mental health, role dynamics, and overall family quality of life would provide a more holistic perspective and inform family-centered support strategies. Addressing these research gaps is crucial to ensuring that families receive the comprehensive, evidence-based support they need.

## Limitation

A significant limitation of this review is the reliance on only two studies that address the impact of raising a child with achondroplasia on parental outcomes. This scarcity of focused peer-reviewed research limits the breadth of evidence available and reduces the generalizability of the findings. As a result, the conclusions drawn must be interpreted with caution, particularly in relation to diverse family structures, cultural backgrounds, and healthcare contexts that may not have been represented. Additionally, the heterogeneity of the available studies, in terms of their methodologies, sample sizes, and geographic contexts, complicates the synthesis of findings and may introduce biases. Therefore, the review’s conclusions are based on fragmented evidence, limiting the findings’ generalizability and robustness.

Another notable limitation is the lack of detailed demographic information about parent participants in the included studies. In particular, Silibello et al. (25) provide minimal insight into the characteristics of the subgroup of parents of children with achondroplasia. This restricts the ability to evaluate how factors such as socioeconomic status, educational background, or cultural context may influence parental experiences and outcomes. The absence of such data limits the depth of critical analysis and reduces the generalizability of findings across diverse family contexts. Future studies should prioritize including and reporting detailed participant demographics to enhance interpretability and relevance.

This underscores the need for further empirical research across broader and more diverse populations to better understand the range and variability of parental experiences in raising children with achondroplasia.
